# Impact of Park Redesign and Renovation on Children’s Quality of Life

**DOI:** 10.21203/rs.3.rs-4745012/v1

**Published:** 2024-08-13

**Authors:** Houlin Hong, Hanish Kodali, Ann Dunlap, Katarzyna Wyka, Lorna E Thorpe, Kelly R Evenson, Terry T-K Huang

## Abstract

Despite increasing interest in the role of parks on children’s health, there has been little empirical research on the impact of park interventions. We used a quasi-experimental pre-post study design with matched controls to evaluate the effects of park redesign and renovation on children’s quality of life (QoL) in underserved neighborhoods in New York City, with predominantly Hispanic and Black populations. Utilizing longitudinal data from the Physical Activity and Redesigned Community Spaces (PARCS) Study, we examined the parent-reported QoL of 201 children aged 3–11 years living within a 0.3-mile radius of 13 renovated parks compared to 197 children living near 11 control parks before and after the park intervention. QoL was measured using a modified version of the KINDL questionnaire, a health-related QoL scale that assessed children’s physical and emotional well-being, self-esteem, and well-being in home, peer, and school functioning. Linear mixed regression model was used to examine the difference in difference (DID) between the intervention vs. control group for QoL. We found a significant differential improvement in the physical well-being subscale of KINDL in the intervention vs. control group (DID = 6.35, 95% Confidence Interval [CI] = 0.85-11,85, p = 0.024). The effect was particularly strong among girls (DID = 7.88, p = 0.023) and children of the lowest socio-economic background (p < 0.05). No significant DID was found in other KINDL domains. Our study indicated a beneficial impact of improving park quality on the physical well-being of children residing in underserved neighborhoods. These findings lend support for investments in neighborhood parks to advance health equity.

## Introduction

The term "built environment" describes the physical settings constructed by humans, encompassing a variety of structures such as buildings, roads, sidewalks, parks, and various other infrastructures. These elements collectively form the spaces where individuals live, work, and play [1, 2]. The way these environments are designed and built plays a significant role in influencing public health, social interactions, and health-related quality of life (QoL) for individuals. This is especially true for children whose QoL is deeply impacted by the nature of their surrounding environments [3]. QoL represents a person's subjective evaluation of their well-being and life satisfaction. It is shaped by numerous factors such as culture, individual values, personal aspirations, societal expectations, and everyday challenges and experiences [4].

Increasing evidence has highlighted the crucial role that the built environment plays, especially parks, in children's health and well-being [5–8]. Research suggests that access to open spaces, both in terms of size and availability, may have positive effects on children's health-related QoL across different ages [9–11]. These findings have been shown in high-income and low- and middle-income countries, where limited access to green spaces is often linked to emotional and behavioral problems in children as well as a higher risk of poor health [12–15]. Research has also shown a correlation between the presence of green spaces with larger and more tree coverage and lower rates of childhood obesity and health-related QoL [10].

However, studies to date have not yielded consistent findings. For instance, the presence of neighborhood parks was not associated with well-being among 5th -grade children in Canada [16]. Furthermore, a study in Norway showed that parks, playgrounds, and sports fields in the neighborhood were surprisingly associated with more depressive symptoms and negative feelings, although this association was likely confounded by population density [17]. Indeed, a recent systematic review indicated that, to date, there is only a moderate level of evidence linking parks and green spaces to children's QoL [5].

One reason for the inconsistent findings may be because the quality, not just quantity or proximity, of parks can influence their impact on children's well-being [8, 16, 18]. This is particularly relevant in underserved communities, where the quality of parks may more often be poor compared to other areas [19]. To date, there have been few studies that sought to examine the impact of improving park quality on children's well-being, especially in underserved communities. Thus, using a quasi-experimental pre-post design with a matched control group, this study examined the impact of citywide park redesign and renovation on the health-related QoL of predominantly Black and Latino children in underserved neighborhoods in New York City.

### Methods

## Study Context

The data used in this study came from the Physical Activity and Redesigned Community Spaces (PARCS) Study [20], which examined the impact of citywide park redesign and renovation implemented through the Community Parks Initiative (CPI),[21] a $318-million park equity investment by the New York City (United States) Department of Parks and Recreation that led to the redesign and renovation of 67 small parks in low-income neighborhoods [20, 21]. A park was deemed eligible for CPI based on meeting at least two of the following three criteria: 1) a neighborhood poverty rate of 20% or higher; 2) a neighborhood population growth of 25% or higher between 2000–2010; or 3) a neighborhood population density of 110 people or more per acre. In addition, to be eligible, selected parks must not have received more than $250,000 in investment in the prior two decades. The intervention led to improved park features, such as more seating and shaded areas, more trees and vegetation, restored lawns, renovated ball courts and playground equipment, soft surfaces for children's play areas, and improved aesthetics throughout the parks.

The PARCS Study was approved by the Institutional Review Board of the City University of New York (#2016 – 0248). Parents provided written consent prior to enrollment into the study. This study followed the Standards for Quality Improvement Reporting Excellence (SQUIRE) v.2.0 guidelines for quality improvement studies [22].

## Study Sample and Procedure

This study was conducted from 2017 to 2022 [20]. As construction at some CPI sites had commenced prior to this study, we included 24 parks in this specific evaluation: 13 intervention parks that underwent redesign and renovation and were closed for a duration of one to two years and 11 parks that were selected as control parks from the same CPI eligibility list that were not due for renovation during the study period. Intervention and control parks were matched on key socio-demographic characteristics (i.e., percent adults, race/ethnicity, poverty rate) among residents living within the 0.3-mile buffer of each park. Parents or caregivers with children between the ages of 3 and 11 years were recruited into the study. To be eligible, families needed to reside within the 0.3-mile radius around the intervention and control parks (i.e., park neighborhood). They had to speak either English, Spanish, or Chinese, and recruitment and retention strategies have been described previously [23]. Parents or caregivers filled out the study survey at baseline pre-renovation and at follow-up, on average between 3 months to 1 year after park renovation [20]. Participants had the option to fill out the survey electronically or on paper.

## Primary Outcome

The primary outcome of the present study was the modified version of the KINDL questionnaire. KINDL is a validated tool used to assess health-related QoL in children and adolescents [24, 25]. A parent or caregiver completed KINDL to assess their child's QoL in the past 30 days. The questionnaire consisted of six subscales on children's physical well-being, emotional well-being, self-esteem, family relationships, friend relationships, and school functioning. Response options for each question were never (1), seldom (2), sometimes (3), often (4), and all the time (5). Each subscale comprised four questions, and the total score was calculated by summing all the items. The subscales and the total score were then normalized to a range of 0 to 100 according to the published protocol [25]. A higher score represented a better QoL. The KINDL is widely used across diverse child populations and has high internal consistency (Cronbach's alphas = 0.76–0.95) [24, 26, 27]. Given that previous studies were conducted in populations different from our study population, factor analysis was conducted to evaluate the factor structure and internal consistency of the questionnaire in our specific population [26, 28–33].

## Socio-demographic variables

Socio-demographics were collected from the parents at study enrollment and included child age, gender (male, female), and ethnicity (Hispanic, Non-Hispanic Black, and Non-Hispanic Other). Parental characteristics included education attainment (high school or less vs. some college or more), employment status (employed or self-employed vs. not employed, including homemaker, student, unemployed, and retired), marital status (married or living with a partner vs. single, separated or widowed), public housing status (living or not in public housing), and annual household income ($20,000 or more vs. less than $20,000).

## Statistical Analysis

Confirmatory factor analysis was performed to analyze the factor structure of KINDL questionnaire in our sample, and internal consistency was assessed using Cronbach's alpha. Descriptive statistics were computed for KINDL subscales and the total score. Chi-square tests were used to compare demographic characteristics between the intervention and control groups at baseline. Unadjusted and adjusted (for any baseline demographic differences between groups) linear mixed regression models were used to examine the impact of park renovations on child QoL. Separate models for each KINDL subscale and total score included fixed effects of time (baseline, follow-up), group (intervention, control), their interaction in all cases, and as appropriate, demographic covariate(s) in adjusted models. The primary estimates of interest were difference-in-difference (DID) effects, representing the difference between changes in the intervention vs. control groups before and after the improvement intervention in each KINDL domain. Parks were treated as a random effect to account for clustering observations in study park neighborhoods.

To assess whether park renovations differentially impacted socio-demographic subgroups, stratified analyses were conducted for KINDL subscales that indicated a significant differential change in QoL in the overall sample. The strata examined included child gender and ethnicity, parental education, employment, household income, marital status, and public housing status.

Multiple imputations (MI) were performed to address missing data [34]. First, we imputed missing socio-demographics using other socio-demographic variables. Subsequently, we imputed KINDL follow-up data 40 times using the monotone imputation method, with baseline KINDL and study group (intervention, control) as imputation input. Rates of missing data for socio-demographics ranged from 0.25% for housing status to 10.8% for income; follow-up data was imputed for 55% of the sample. The total number of cells missing, including all demographic variables and outcome variables at baseline and follow-up was 6%. Sensitivity analyses were conducted to compare baseline characteristics of the complete-case sample vs. the sample with missing follow-up KINDL, and to compare regression results based on complete cases vs. imputed data.

All statistical analyses were performed using SAS v9.4 (Cary, NC), with the significance level set at 0.05.

### Results

### Sample characteristics

The sample included 398 children, with 201 in the intervention group and 197 in the control group. Children were, on average, 5.8 years old (SD = 1.7); half were female (51.8%), Hispanic (52.3%), and slightly over a quarter were Non-Hispanic Black (28.4%). Public housing residents accounted for nearly two-fifths of the sample (36.9%). Approximately half of parents/caregivers completed some college or more (49.5%), were not employed (49.8%), and reported an annual household income of <$20,000 (51.5%). There were no significant differences between the groups except that participants in the control groups were more likely to be public housing residents (44.7% vs. 29.4%, $p < 0.001$) (Table 1).

Table 1
Socio-demographic characteristics of the study sample.

| | **Overall** | **Control** | **Intervention** |
|---|---|---|---|
| | **n = 398** | **n = 197** | **n = 201** |
| **Child Age** | | | |
| Mean (SD) | 5.76 (1.68) | 5.84 (1.68) | 5.69 (1.68) |
| Median (Q1, Q3) | 6.00 (4.00, 7.00) | 6.00 (5.00, 7.00) | 6.00 (4.00, 7.00) |
| | **N (%)** | **N (%)** | **N (%)** |
| **Child Gender** | | | |
| Female | 206 (51.8) | 102 (51.8) | 104 (51.7) |
| Male | 192 (48.2) | 95 (48.2) | 97 (48.3) |
| **Child Ethnicity** | | | |
| Hispanic | 208 (52.3) | 109 (55.3) | 99 (49.3) |
| Non-Hispanic Black | 113 (28.4) | 47 (23.9) | 66 (32.8) |
| Non-Hispanic Other | 77 (19.4) | 41 (20.8) | 36 (17.9) |
| **Parental Education** | | | |
| High School or Less | 201 (50.5) | 98 (49.8) | 103 (51.2) |
| Some College or More | 197 (49.5) | 99 (50.2) | 98 (48.8) |
| **Parental Employment Status** | | | |
| Not Employed | 198 (49.8) | 97 (49.3) | 101 (50.2) |
| Employed | 200 (50.2) | 100 (50.7) | 100 (49.8) |
| **Parental Marital Status** | | | |
| Married/Live with a Partner | 170 (42.7) | 78 (39.6) | 92 (45.8) |
| Unmarried/Divorced/Widowed | 228 (57.3) | 119 (60.4) | 109 (54.2) |
| **Public Housing Status** | | | |
| Non-Public Housing Resident | 251 (63.1) | **109 (55.3)** | **142 (70.7)** |
| Public Housing Resident | 147 (36.9) | **88 (44.7)** | **59 (29.3)** |
| **Annual Household Income** | | | |
| ≥$20,000 | 193 (48.5) | 86 (43.7) | 107 (53.2) |

Note: Bolded numbers represent statistically significant difference between the intervention and control group ($p < 0.001$).

| | Overall | Control | Intervention |
|---|---|---|---|
| | **n = 398** | **n = 197** | **n = 201** |
| **Child Age** | | | |
| <$20,000 | 205 (51.5) | 111 (56.3) | 94 (46.8) |
| Note: Bolded numbers represent statistically significant difference between the intervention and control group ($p < 0.001$). | | | |

## Factor analysis of KINDL

The original KINDL questionnaire has four questions for each of the six subscales, and a total score is generated by summing scores for all 24 questions. Confirmatory factor analysis of our data generated a factor structure consistent with the original KINDL questionnaire; however, six items had insufficient factor loading (< 0.4) for the predefined subscales. Therefore, we reduced the overall scale from 24 to 18 items, resulting in two to four items per subscale.Cronbach's alpha for the modified KINDL was satisfactory (overall = 0.88, physical well-being = 0.74, emotional well-being = 0.63, self-esteem = 0.82, family relationships = 0.76, friend relationships = 0.77, school functioning = 0.78) (Table 2).

Table 2
Factor loadings for the modified KINDL scale.

| Items: My child… | Factors | | | | | |
|---|---|---|---|---|---|---|
| | Physical Well-being | Emotional Well-being | Self-Esteem | Family Relationships | Friend Relationships | School Functioning |
| felt ill | 0.83 | | | | | |
| had a headache or tummy-ache | 0.85 | | | | | |
| was tired and worn-out | 0.52 | | | | | |
| had fun and laughed a lot | | 0.66 | | | | |
| didn’t feel much like doing anything | | 0.48 | | | | |
| felt alone | | 0.52 | | | | |
| felt scared or unsure of him/herself | | 0.57 | | | | |
| was proud of him/herself | | | 0.79 | | | |
| felt on top of the world | | | 0.71 | | | |
| felt pleased with him/herself | | | 0.81 | | | |
| had lots of good ideas | | | 0.69 | | | |
| got on well with us as parents | | | | 0.86 | | |
| felt fine at home | | | | 0.72 | | |
| played with friends | | | | | 0.61 | |
| was liked by other kids | | | | | 0.78 | |
| got along well with his friends | | | | | 0.78 | |
| coped well with the assignments | | | | | | 0.72 |

Note: The overall scale was reduced from 24 to 16 items following confirmatory factor analysis (Cronbach’s alpha of the modified scale = 0.88, physical well-being = 0.74, emotional well-being = 0.63, self-esteem = 0.82, family relationships = 0.76, friend relationships = 0.77, school functioning = 0.78).

| Items: My child… | Factors | | | | | |
|---|---|---|---|---|---|---|
| | Physical Well-being | Emotional Well-being | Self-Esteem | Family Relationships | Friend Relationships | School Functioning |
| set in nursery/school | | | | | | |
| enjoyed the nursery/school | | | | | | 0.81 |

Note: The overall scale was reduced from 24 to 16 items following confirmatory factor analysis (Cronbach's alpha of the modified scale = 0.88, physical well-being = 0.74, emotional well-being = 0.63, self-esteem = 0.82, family relationships = 0.76, friend relationships = 0.77, school functioning = 0.78).

## Changes in QoL following park renovations

Mean scores for KINDL over time in the intervention and control groups are shown in Table 3. Linear mixed regression model showed that the mean physical well-being score increased significantly from baseline to post-intervention in the intervention group (Δ = 7.25, SE = 2.10, p < 0.001), while the control group showed no change (Δ = 0.90, SE = 1.80, p = 0.616). The differential improvement in the intervention vs. control group was statistically significant (DID = 6.35, 95%CI = 0.85-11,85, p = 0.024) (Table 4). No statistically significant differences were found in any other KINDL subscale or the total KINDL score (Table S1). These findings did not change when public housing status was included in the models as a covariate (data not shown).

Table 3
Mean KINDL scores over time.

| | Overall (n = 398) | | Control (n = 201) | | Intervention (n = 197) | | Between group p value at baseline |
|---|---|---|---|---|---|---|---|
| Subscales | Baseline | Follow-up | Baseline | Follow-up | Baseline | Follow-up | |
| Physical Well-being | 79.68 (19.49) | 83.79 (15.57) | 80.53 (19.50) | 81.43 (15.81) | **78.84 (19.44)** | **86.10 (14.98)** | 0.34 |
| Emotional Well-being | 86.76 (14.09) | 86.07 (14.66) | 86.85 (14.13) | 85.79 (14.82) | 86.68 (14.05) | 86.34 (14.50) | 0.76 |
| Self-Esteem | 78.12 (19.07) | 76.82 (18.46) | 78.98 (17.83) | 76.47 (17.85) | 77.28 (20.17) | 77.17 (19.02) | 0.37 |
| Family Relationships | 87.47 (18.29) | 88.38 (15.19) | 87.30 (18.62) | 88.98 (14.77) | 87.64 (17.96) | 87.79 (15.57) | 0.84 |
| Friend Relationships | 80.24 (18.66) | 80.10 (18.94) | 81.18 (17.23) | 82.12 (17.68) | 79.31 (19.92) | 78.12 (19.89) | 0.32 |
| School Functioning | 81.20 (22.24) | 79.06 (22.49) | 82.35 (20.65) | 82.59 (20.53) | 80.09 (23.62) | 75.63 (23.74) | 0.32 |
| Total Score | 82.00 (13.04) | 82.10 (10.98) | 82.69 (12.23) | 82.20 (10.37) | 81.32 (13.79) | 81.97 (11.80) | 0.73 |

Note: Statistics represent normalized means (standard deviations, SD) with a possible range of 0–100 for each subscale and the total score. Bolded numbers represent statistically significant (p < 0.001) within-group change over time.

Table 4
Difference-in-difference (DID) effects for change in KINDL physical well-being in the total sample and stratified by socio-demographic subgroups.

| | Control Group | | | Intervention Group | | | DID (Intervention - Control) | |
|---|---|---|---|---|---|---|---|---|
| | **Baseline Mean (SE)** | **Change Post-Pre (SE)** | **p-value** | **Baseline Mean (SE)** | **Change Post-Pre (SE)** | **p-value** | **DID (SE)** | **p-value** |
| **Total Sample** | 80.53 (1.25) | 0.9 (1.80) | 0.616 | 78.84 (1.24) | 7.25 (2.10) | **< 0.001** | 6.35 (2.79) | **0.024** |
| **Stratified Analyses** | | | | | | | | |
| **Child Gender** | | | | | | | | |
| Male | 80.64 (1.79) | 0.33 (2.57) | 0.898 | 81.33 (1.77) | 5.26 (3.13) | 0.095 | 4.93 (3.81) | 0.197 |
| Female | 80.41 (1.75) | 1.52 (2.43) | 0.532 | 76.18 (1.74) | 9.39 (2.47) | **< 0.001** | 7.88 (3.44) | **0.023** |
| **Child Ethnicity** | | | | | | | | |
| Hispanic | 79.13 (1.74) | 1.14 (2.56) | 0.656 | 78.85 (1.84) | 7.70 (2.80) | **0.006** | 6.56 (3.79) | 0.084 |
| Non-Hispanic Black | 82.45 (2.50) | 2.47 (3.69) | 0.504 | 81.31 (2.11) | 6.23 (3.44) | 0.071 | 3.76 (4.77) | 0.431 |
| Non-Hispanic Other | 82.05 (2.60) | -1.53 (3.43) | 0.655 | 74.31 (2.74) | 7.91 (4.08) | 0.054 | 9.44 (5.12) | 0.066 |
| **Parental Education** | | | | | | | | |
| High School or Less | 80.19 (1.82) | -2.18 (2.66) | 0.414 | 78.41 (1.79) | 6.20 (2.67) | **0.021** | 8.38 (3.69) | **0.024** |
| Some College or More | 80.86 (1.71) | 3.95 (2.44) | 0.106 | 79.29 (1.71) | 8.36 (2.98) | **0.006** | 4.41 (3.71) | 0.236 |
| **Parental Employment Status** | | | | | | | | |
| Not Employed | 80.33 (1.79) | 1.14 (2.71) | 0.674 | 77.61 (1.76) | 8.67 (3.11) | **0.006** | 7.53 (3.80) | **0.048** |
| Employed | 80.50 (1.76) | 0.63 (2.56) | 0.807 | 80.15 (1.76) | 5.27 (2.71) | 0.053 | 4.64 (3.67) | 0.207 |
| **Parental Marital Status** | | | | | | | | |
| Single/Widowed/Divorced | 82.33 (1.92) | 1.89 (2.83) | 0.504 | 77.98 (1.77) | 10.56 (2.76) | **< 0.001** | 8.67 (3.86) | **0.025** |

Note. Model estimates and p-values are from linear mixed regression models performed on imputed datasets. Statistically significant results ($p < 0.05$) are shown in bold.

| | Control Group | | | Intervention Group | | | DID (Intervention - Control) | |
|---|---|---|---|---|---|---|---|---|
| | Baseline Mean (SE) | Change Post-Pre (SE) | p-value | Baseline Mean (SE) | Change Post-Pre (SE) | p-value | DID (SE) | p-value |
| Married or Live with Partner | 79.23 (1.64) | -0.09 (2.40) | 0.972 | 79.46 (1.72) | 4.02 (3.01) | 0.184 | 4.10 (3.72) | 0.271 |
| **Public Housing Status** | | | | | | | | |
| Public Housing Resident | 80.08 (1.65) | 1.55 (2.49) | 0.534 | 78.24 (1.45) | 7.13 (2.34) | **0.003** | 5.58 (3.44) | 0.106 |
| Non-Public Housing Resident | 81.06 (1.93) | -0.11 (2.83) | 0.970 | 80.38 (2.37) | 7.28 (3.55) | **0.041** | 7.39 (4.38) | 0.092 |
| **Annual Household Income** | | | | | | | | |
| ≥ 20,000 | 79.65 (1.81) | 2.04 (2.79) | 0.466 | 81.46 (1.63) | 5.20 (2.68) | 0.054 | 3.16 (3.73) | 0.398 |
| < 20,000 | 81.25 (1.73) | -0.01 (2.69) | 0.998 | 76.00 (1.89) | 9.61 (2.98) | **0.002** | 9.62 (3.79) | **0.012** |
| Note. Model estimates and p-values are from linear mixed regression models performed on imputed datasets. Statistically significant results ($p < 0.05$) are shown in bold. | | | | | | | | |

We further conducted stratified analysis by intervention status for the physical well-being subscale. Within the intervention group, significant increases from baseline to follow-up in physical well-being were found among girls (Δ = 9.39, SE = 2.47, p < 0.001), Hispanic children (Δ = 7.70, SE = 2.80, p = 0.006), children of parents with both lower and higher education attainment (high school or less: Δ = 6.20, SE = 2.67, p = 0.021; some college or more: Δ = 8.36, SE = 2.98, p = 0.006), children of parents not employed (Δ = 8.67, SE = 3.11, p = 0.006), children from households with an annual income <$20,000 (Δ = 9.61, SE = 2.98, p = 0.002), children of single, widowed or divorced parents (Δ = 10.56, SE = 2.76, p < 0.001), and those residing in both public housing (Δ = 7.13, SE = 2.34, p = 0.003) and non-public housing (Δ = 7.28, SE = 3.55, p = 0.041). No significant changes were observed in these demographic subgroups within the control group.

DID analyses indicated that the intervention group had a significant differential improvement in physical well-being relative to that in the control group among girls (DID = 7.88, 95%CI = 1.14–14.62, p = 0.023), children of parents with high school or less (DID = 8.38, 95%CI = 1.15–15.61, p = 0.02), children with parents not employed (DID = 7.53, SE = 0.08–14.98, p < 0.05), children in households with annual income <$20,000 (DID = 9.62, SE = 2.19–17.05, p = 0.01), and children of single, widowed or divorced parents (DID = 8.67, SE = 1.10-16.24 p = 0.03).

Sensitivity analyses

When comparing the complete-case sample to the sample with missing follow-up KINDL data, we found no significant differences in baseline QoL measures, but the latter had lower household income (44.4% vs. 56.5% <$20,000, p = 0.023) and higher non-employment (56.0% vs, 41.9% non-employment, p = 0.006). However, we found similar regression results between the complete-case sample (n = 77 and 103 in the intervention and control groups, respectively) and the full sample (Tables S2 and S3).

## Discussion

To our knowledge, this is one of the first intervention studies on park quality improvement and its impact on children's QoL. Our study found a significant improvement in the physical well-being dimension of QoL among children living in underserved neighborhoods following park renovation compared to those living in control neighborhoods without park renovation.

QoL has increasingly been recognized as a crucial health outcome, reflecting a broader understanding of health that goes beyond traditional clinical measures [35]. Despite this growing recognition, research on QoL in children has remained relatively understudied, leaving a significant gap in our understanding of how best to support the well-being of younger populations [5]. The KINDL questionnaire is a widely used tool for assessing QoL in children across diverse settings [28–33]; however, there is still a scarcity of studies focusing on QoL in children and built environment using KINDL questionnaire and effective strategies to enhance QoL in children. This study addressed this gap by exploring the potential of environmental interventions by demonstrating how changes in the environment can positively impact children's QoL.

Our findings echo previous research emphasizing the influence of the built environment on children's physical health and well-being [36–40]. Parks may directly contribute to better health outcomes in children, and the availability of greenspaces has shown to play a crucial role in in promoting physical aspects of health-related QoL among urban youth. For instance, higher green coverage in a community and proximity to green spaces were associated with greater physical and mental health in children [7, 40, 41]. One mechanism for such associations might be that green spaces extend more opportunities for engagement in active play and physical activity [6]. Children with limited park access have been shown to be less likely to engage in physical activity on a weekly basis [6, 7, 42]. Renovated CPI parks often feature new or improved play equipment, safer surfaces, and better-maintained green areas, which may contribute to encouraging more active play.

Other mechanisms that might explain the relationship between parks and health-related QoL including the potential for green spaces to mitigate against the effect of adverse environmental exposures, such as air pollution, on the risk for ill health [36]. Additionally, green spaces may promote social development within a neighborhood by promoting social interaction, autonomy, and self-esteem among children [11, 43]. However, while a positive social environment could be directly linked to physical well-being [45], we did not find effects of neighborhood park renovation on other KINDL domains, such as self-esteem or family and friend relationships. To the best of our knowledge, only one study has demonstrated a significant association between green space use and self-esteem and friend relationships based on the KINDL questionnaire [43]. One potential explanation for the discrepancy between this study and our findings could be the sample demographics. Our sample was predominantly Hispanic and Black from low-income families in New York City, with an average age of 5.8 years. On the contrary, the study by McCracken et al [43] was conducted in urban Scotland with an average age of 9.7 years, and was based on a cross-sectional design. A third possible explanation might be that the effect of having a quality park in the neighborhood differs from actual park use. Thus, the social catalyst aspect of parks warrants further research.

The subgroup analyses uncovered additional insights. Notably, we found that the benefit of park quality improvement on physical well-being was stronger in girls than boys. Past research has shown that boys may be more likely to use parks than girls [46, 47], which may be explained, in part, by other research showing that perceived safety is associated with park use [48]. It is likely that CPI has improved the sense of safety at study parks since park usage increased more among females than males at CPI parks [49]. Based on this, it is plausible that the link between park improvement and physical well-being is, in turn, more pronounced among girls.

Our subgroup analyses also showed that the park improvement effect on physical well-being was stronger among children from the lowest socio-economic status, specifically those living with parents who were single/divorced/widowed, less educated, not employed, or had an annual household income <$20,000. This finding substantiates the equity lens at the heart of CPI. Quality parks could serve as crucial public amenities that compensate for a lack of access to recreational gyms or organized sports, thus playing a pivotal role in mitigating health disparities. Enhanced physical well-being among children can lead to reduced incidence of chronic diseases and better overall health outcomes. One study showed that children living below the federal poverty level were 48% more likely to lack a park in their neighborhood, compared to wealthier children [42]. There is also evidence that high-quality parks are more frequently found in higher-income cities [50]. Our findings suggest that interventions such as CPI can play an important role in addressing health disparities in low socio-economic and minoritized communities.

The quasi-experimental design with matched controls makes this study an important and unique contribution to the literature on the role of parks in children's QoL. While missingness at follow-up was a limitation and might have introduced bias, results from imputed and complete-case analyses yielded the same conclusion. In this study, we were not able to examine the role of specific park features as this was beyond the scope of this paper. However, this warrants future research. Finally, despite having a diverse sample, this study was based on convenience sampling and may not be representative of the underlying population. Therefore, generalization of these results should be cautioned.

In conclusion, we found that park redesign and renovation in low-income neighborhoods had a positive effect on the physical well-being dimension of QoL among children living in close proximity to renovated parks compared to those living near parks that were not renovated. The effect was even stronger among children with the lowest socio-economic profile. This study expands our understanding of how built environment interventions, particularly regarding parks, can benefit the well-being of children and help reduce health disparities in lower socio-economic settings and minority communities. Insights from this study can inform future programs and policies that integrate urban planning with public health and health equity.

## Declarations

## Funding

This study was supported by the National Cancer Institute (R01CA206877), New York State Health Foundation (#16-04236), Robert Wood Johnson Foundation (E4A Program Grant #76473), and Bryant Park Corporation. Additional funding support was provided by a grant from the Centers for Disease Control and Prevention (U48DP006396).

### Figures

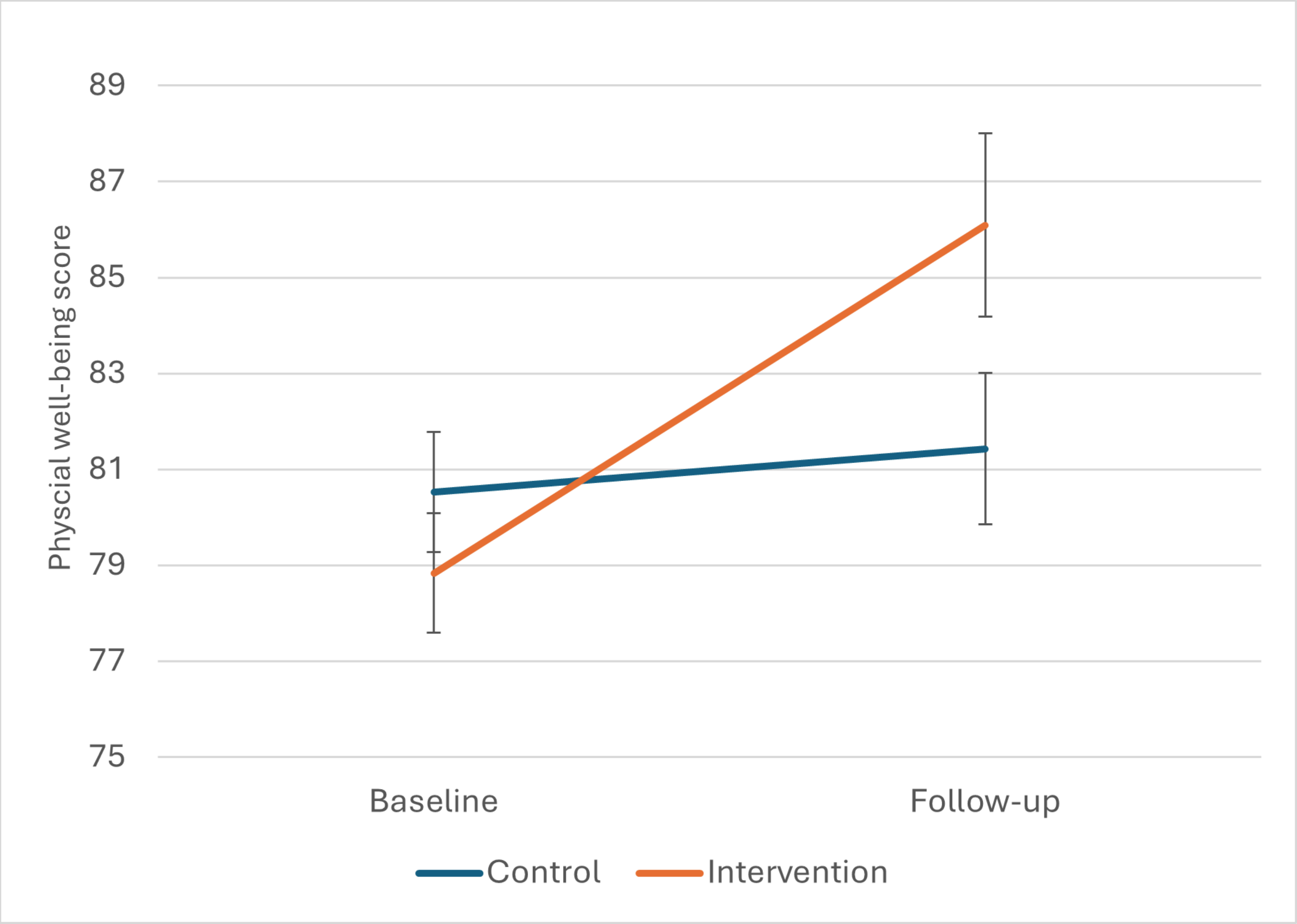

**Figure 1**

Changes in KINDL physical well-being over time in intervention and control groups.

Note: The error bars in the graph represent standard errors.

### Supplementary Files

This is a list of supplementary files associated with this preprint. Click to download.

- SupplementarytableJUH.docx
